# Perfused Platforms to Mimic Bone Microenvironment at the Macro/Milli/Microscale: Pros and Cons

**DOI:** 10.3389/fcell.2021.760667

**Published:** 2022-01-03

**Authors:** Maria Veronica Lipreri, Nicola Baldini, Gabriela Graziani, Sofia Avnet

**Affiliations:** ^1^ Department of Biomedical and Neuromotor Sciences, University of Bologna, Bologna, Italy; ^2^ Biomedical Science and Technologies Lab, IRCSS Istituto Ortopedico Rizzoli, Bologna, Italy; ^3^ Laboratory for NanoBiotechnology (NaBi), IRCCS Istituto Ortopedico Rizzoli, Bologna, Italy

**Keywords:** bone, perfused model, *in vitro*, macroscale, microscale, microfluidics, 3D models

## Abstract

As life expectancy increases, the population experiences progressive ageing. Ageing, in turn, is connected to an increase in bone-related diseases (i.e., osteoporosis and increased risk of fractures). Hence, the search for new approaches to study the occurrence of bone-related diseases and to develop new drugs for their prevention and treatment becomes more pressing. However, to date, a reliable *in vitro* model that can fully recapitulate the characteristics of bone tissue, either in physiological or altered conditions, is not available. Indeed, current methods for modelling normal and pathological bone are poor predictors of treatment outcomes in humans, as they fail to mimic the *in vivo* cellular microenvironment and tissue complexity. Bone, in fact, is a dynamic network including differently specialized cells and the extracellular matrix, constantly subjected to external and internal stimuli. To this regard, perfused vascularized models are a novel field of investigation that can offer a new technological approach to overcome the limitations of traditional cell culture methods. It allows the combination of perfusion, mechanical and biochemical stimuli, biological cues, biomaterials (mimicking the extracellular matrix of bone), and multiple cell types. This review will discuss macro, milli, and microscale perfused devices designed to model bone structure and microenvironment, focusing on the role of perfusion and encompassing different degrees of complexity. These devices are a very first, though promising, step for the development of 3D *in vitro* platforms for preclinical screening of novel anabolic or anti-catabolic therapeutic approaches to improve bone health.

## 1 Introduction

The ageing of global population is increasing steadily, thanks to the progress in medicine and therapy. However, the increase of population age also brings an increase in the prevalence of common age-related diseases, including cancer, arthritis and osteoporosis, along with falls-induced fractures^1^. Musculoskeletal condition affects about 126.6 million Americans (one each two adults), resulting in an estimated $213 billion annual economic burden for treatment, care and lost wages. This societal and economic burden is increasing over time (National Academies of Sciences, 2020 Apr 21). In this scenario, there is an urgent need for more reliable pre-clinical models that can fully recapitulate bone tissue characteristics for the study of bone physiology and physiopathology, and for drug screening, with particular regard to bone-related diseases in the elders.

To date, in this field, many challenges are yet to be addressed because bone is a highly complex tissue and so its modelling has a significant degree of complexity. The skeleton is in fact an extremely specialized and dynamic organ that undergoes continuous regeneration, namely “bone remodelling”, a dynamic and delicate equilibrium between bone resorption and bone deposition. These are regulated by bone cells: the osteoblasts, that have a mesenchymal origin and that deposit collagen type I and the mineralized matrix; the osteocytes, the most differentiated form of osteoblasts, and that are embedded in the mineralized bone matrix; the osteoclasts, that have a hematopoietic origin, and that degrade bone via secretion of acid and proteolytic enzymes. Osteoblast-osteoclast coupling, directed by osteocytes, is the main actor of the bone remodelling process since osteoblasts are responsible for bone deposition and osteoclasts for bone resorption. In addition, bone is a highly vascularized multicellular tissue surrounded by an extracellular matrix (ECM) (Gentili and Cancedda, 2009; Scheinpflug et al., 2018; Mckee et al., 2019), and provides both mechanical functions, i.e., locomotion and protection of internal organs, and metabolic functions, i.e., mineral homeostasis and haematopoiesis (Scheinpflug et al., 2018). The ECM is a composite material constituted by an inorganic (∼60 wt%) and an organic (∼30 wt%) phase. Bone apatite, the mineralized (inorganic) phase of bone, is composed by ion-substituted nanocrystalline carbonated hydroxyapatite (HA) and is responsible for the high mechanical stability and load-bearing properties of the ECM (Zaidi, 2007; J. L. Brown, 2013; Alford et al., 2015; Mckee et al., 2019). Flexibility, instead, is provided by the organic phase mainly formed by type I collagen, non-collagenous glycoproteins, hyaluronan, proteoglycans and growth factors secreted by cells (Alford et al., 2015; Mckee et al., 2019).

The gold standard for the study of bone and bone diseases still relies on the use of *in vivo* animal models. However, animal research is an ethical dilemma, and the use of *in vivo* models allows limited possibility to tune and mimic tissue microenvironment, as well as a scarce reproducibility. On the other hand, conventional two dimensional (2D) *in vitro* cell cultures of bone cells, obtained with the addition of pro-osteogenic and -osteolytic growth factors in the culture media, are highly reproducible, fast and ethical, but are poorly predictive of clinical outcomes, as they fail to reproduce the complexity of the dynamic microenvironment of bone. Indeed, in patients, in addition to the local secretion of differentiating factors and by the unique composition of the ECM, bone homeostasis is finely and continuously tuned by variable and multi-axial mechanical loading, and by the coexistence of biochemical cues, like nutrient and oxygen gradients (Shin et al., 2012; Scheinpflug et al., 2018; Sleeboom et al., 2018) that are rarely reproduced in 2D conditions. It is already widely recognised that mechanical loading is a major driver of bone mass and structural adaptation (Turner, 1998; Skerry, 2008; Galea et al., 2017). Several *in vivo* studies have demonstrated that gravitational forces and mechanical loads generated by muscle contractions are essential to stimulate bone remodelling and to maintain high mechanical performance (Petersen et al., 2012; Hao et al., 2013). Most importantly, mechanical stimulation through perfusion-induced fluid shear stress plays a crucial role on bone differentiation and mineralization, vasculogenesis and mechanotransduction (Alfieri et al., 2019). More in details, at the micro-scale, *in vivo* mechanical strains, including fluid shear stress, cyclic stretching, compression, and uniaxial deformation, can strongly modulate bone cell behaviour through the ECM. The ECM allows the transmission of physical forces to the cell cytoskeleton via physical mechanotransduction, activating a signalling cascade, which affects cellular functions such as proliferation, migration, differentiation, and apoptosis (Sikavitsas et al., 2001; Mccoy and O’brien, 2010; Wittkowske et al., 2016). Among these mechanical strains, fluid shear stress is induced by interstitial perfusion, which, in turn, results from pressure gradients produced by vascular and hydrostatic pressure, and ca be induced by mechanical loading (Hillsley and Frangos, 1994; Mccoy and O’brien, 2010; Yao et al., 2012; Wittkowske et al., 2016; Yuste et al., 2021). These are all crucial players of mechanical stimulation of physiological tissue microenvironment (Mccoy and O’brien, 2010; Wittkowske et al., 2016; Yuste et al., 2021) but also allow for increased diffusion of nutrients, metabolites, and oxygen, removal of toxic products or inhibitors of cellular metabolism, thereby preventing the formation of necrotic core areas (Rouwkema et al., 2010; Place et al., 2017). Therefore, static *in vitro* cell cultures are highly limited by the lack of vasculature, which results in a scarce perfusion of cellular nutrients and dispersion of waste cellular products (200 µm). Clearly, these limitations make 2D static *in vitro* models not suitable for clinically relevant bone models.

To better resemble the bone microenvironment, several examples of 3D *in vitro* bone models are available, including the use of spheroids, 3D scaffolds, cell sheets, hydrogels, bioreactors, and microfluidics (Wittkowske et al., 2016; Yuste et al., 2021). Among these, 3D *in vitro* fluidic macroscale (i.e., spinner flasks, rotating wall vessels), milliscale (customized perfusion bioreactors), and microscale systems (i.e., microfluidic devices) appear very promising to overcome the limitations of 2D cultures. These devices allow a fine tuning of dynamic interstitial perfusion (Wittkowske et al., 2016; Yuste et al., 2021), a full understanding of cell-cell and cells-ECM interactions and, overall, a better comprehension of *in vivo* biological mechanisms (Kim et al., 2007; Esch et al., 2015; Arrigoni et al., 2017; Carvalho et al., 2018; Wang et al., 2018; Nokhbatolfoghahaei et al., 2020a). So far, good outcomes have been achieved in reproducing structural, functional, and mechanical properties of tissues using perfused platforms, including lung alveoli and bronchioles (Huh et al., 2010; Ott et al., 2010; Price et al., 2010; Stucki et al., 2015), renal tubules and glomeruli (Humes et al., 1999; Humes et al., 2004; Jang and Suh, 2010; Wilmer et al., 2016), small intestine (Kimura et al., 2008; Imura et al., 2009; Pusch et al., 2011; Schweinlin et al., 2016), liver (Kane et al., 2006; Tsang et al., 2007; Yamada et al., 2007; Domansky et al., 2010; Elbakary and Badhan, 2020) and the blood-brain barrier (Booth and Kim, 2012; Griep et al., 2013). Even though the complexity achieved by these technologies is increasing rapidly, their use in the bone field has been slow to keep up. However, such technologies are extremely promising for tissue/disease modelling and drug screening, for a better prediction on drug efficacy and toxicity, as shown by the very recent literature on their applications in other medical fields (Ahmed et al., 2019), and a quick spread of research on this topic is very likely in the coming years.

In this review, we analysed the state-of-art, limitations and recent breakthrough in the development of perfused micro, milli and macroscale 3D systems in reproducing and modelling bone. We gave particular attention to the impact of perfusion on directing the chemical and physical behaviour of the models and on dictating biological processes.

## 2 *In vitro* Approaches to Mimic Interstitial Fluid Flow: An Overview of Biomechanical Clues

When developing a bone model, several different parameters should be considered depending on the aims of the study and application. Mimicking interstitial fluid flow and the shear stress has particular relevance (Mccoy and O’brien, 2010) but is also challenging due to the bone tissue heterogeneity. In fact, the exact physical (e.g., architecture, porosity) and chemical characteristics (e.g., organic/inorganic composition) of the ECM dictate its permeability to fluids and mechanical features (Mccoy and O’brien, 2010). Morphological and physicochemical characteristics, in turn, influence the response to shear stress (Mccoy and O’brien, 2010).

More in details, interstitial fluids flow through the porous mineral matrix of cancellous and cortical bone determines different extents of shear stress, depending on the pore size (from the micro to the nanoscale): 1) the vascular porosity within the Volkmann canal and Haversian canals (ø∼40 μm, micropores); 2) the lacunae-canaliculi system which are the channel structures within the mineralized bone tissue surrounding osteocytes and their dendritic processes (ø∼0.2 μm); and 3) the sub-micrometric spaces between crystallites of the mineral hydroxyapatite and collagen fibres (∼ø 0.02 μm) (Cowin and Cardoso, 2015; Wittkowske et al., 2016). Mechanical stress is size dependent and is generally higher in smaller vessels. However, vessels shape that is determined by section geometry, surface roughness, and presence of defects is also important. Furthermore, different cell types sense different shear stress levels. For instance, osteocytes reside in interconnected microscale spaces (namely the osteocytes lacunae) (Nicolella et al., 2006)) and are surrounded by a stiff extracellular matrix. This results in a high value of fluid shear stress, which ranges between 0.8 and 3.0 Pa, according to the numerical model by Weinbaum et al. (1994), Wittkowske et al. (2016). In contrast, in growing bone, osteoblasts are not surrounded by calcified bone matrix but are located on the surface of soft osteoid in highly porous regions. In this case, fluid flow and fluid shear stress are lower (shear stress <0.8 Pa) (Liegibel et al., 2004; Mcgarry et al., 2005; Bonewald and Johnson, 2008). A correct estimation of the shear stress sensed by osteoblasts is further complicated by the constant remodelling of the channels that surround them, and by the lack of knowledge regarding the mechanical properties of the soft osteoid (Wittkowske et al., 2016).

In conclusion, to date, in *in vitro* models, dynamic perfusion of the culture system is the most widely and accepted method to resemble the interstitial fluid movement caused by compression and tension, as it exposes cells to perfusion-induced shear stress loading (Mccoy and O’brien, 2010). Depending on the aims of the model, perfusion can be applied to macro-, milli-, and micro-scale systems through passive or active systems. In the following sections, we thoroughly described the different methods that are available to increase the similarity to the *in vivo* fluid flow, at the different scales and with different degrees of complexity.

### 2.1 Macro/Milli Scale Models

At the macroscale, perfusion is influenced both by scaffold composition and architecture, and by the features of the chosen perfusion system.

In terms of scaffold composition and architecture, bone scaffolds must be highly porous since porosity up to 90% facilitates fluid perfusion inside the structure of the scaffold (Abbasi et al., 2020). It also influences mechanical properties, and biological features of bone tissue, by promoting cell adhesion and proliferation (Mccoy and O’brien, 2010; Yeatts and Fisher, 2011; Wittkowske et al., 2016; Zhao et al., 2016). The most long used techniques to obtain porosity in 3D bone scaffolds are: 1) solvent casting/particulate leaching technique, based on the use of substances (porogens) dispersed in a polymer solution and dissolved when the structure is set; 2) foaming gas, based on the use carbon dioxide at high pressure; 3) freeze-drying, based on the removal of water or other solvents under a vacuum, in a frozen sample; 4) phase separation technique, based on thermal separation of a polymer solution into a polymer-rich phase and a solvent-rich phase that is removed by extraction, evaporation, or sublimation. However, these techniques often lack a precise control of the microarchitecture, due to the scarce control over pore shape, size, and interconnectivity (Tu et al., 2003; Hollister, 2005; Janik et al., 2015; Fereshteh, 2018). Additive manufacturing/3D printing is a more recent scaffold fabrication technique which is more reproducible and accurate, and allows controlling geometry at the macro and microscale (Bose et al., 2013). The 3D structure is obtained by a computer-aided design-based model and is formed, layer-by-layer, through the deposition of a powder, liquid, or solid materials. Notably, among these techniques, bioprinting also allows the simultaneous deposition of biomaterials and cells, thus recapitulating both bone microarchitecture and cell distributions of native tissues. For a comprehensive overview of 3D printing techniques, please refer to Moroni et al. (2018).

**FIGURE 1:**
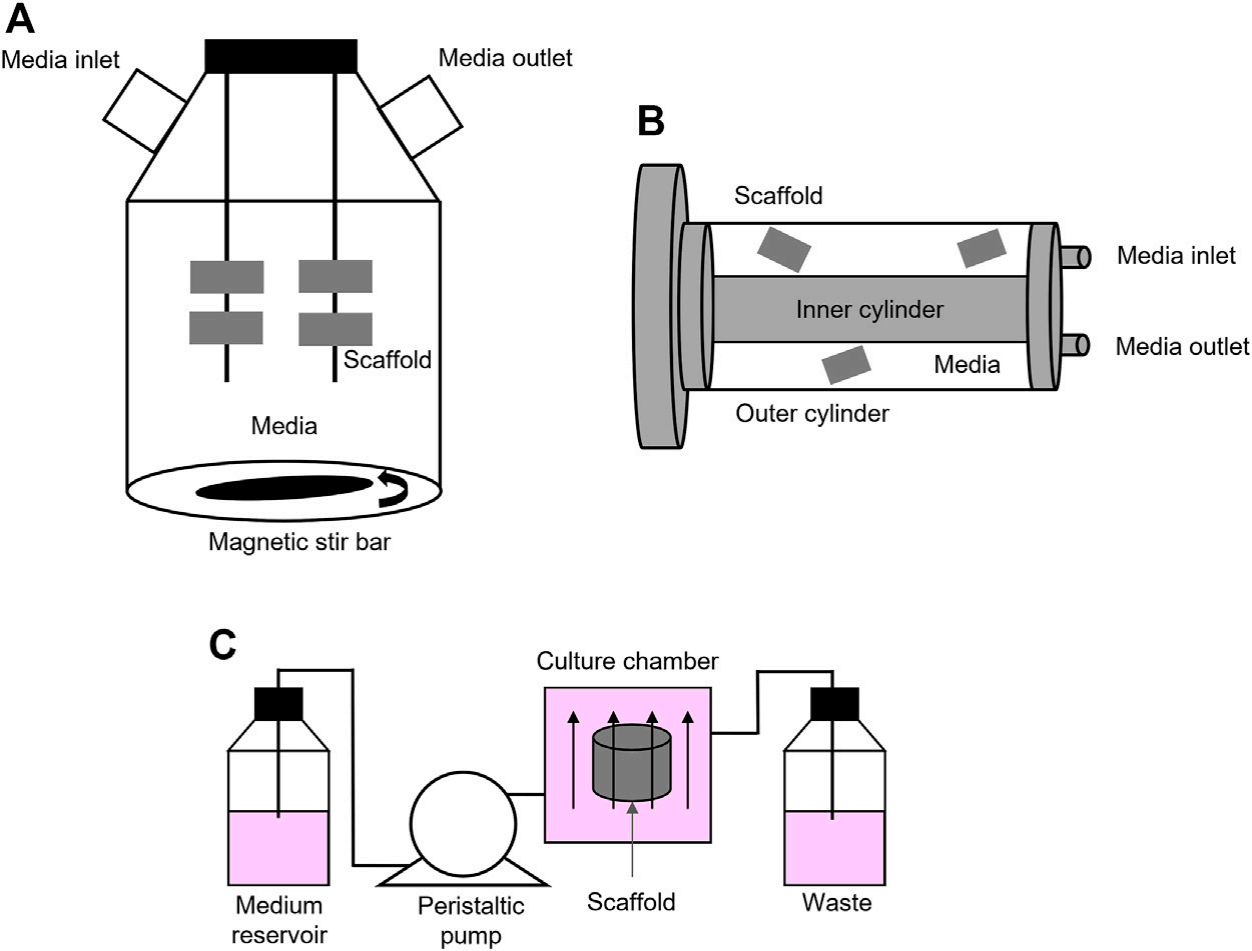
Bioreactors: **(A)** Schematic representation of a spinner flask bioreactor. Bone scaffolds are suspended in stirred circulating media; **(B)** Schematic representation of a rotating bioreactor. Outer cylinder motion allows the circulation of media; **(C)** Schematic representation of a basic perfusion bioreactor system that is formed by a culture media reservoir, a peristaltic pump, a culture chamber, and waste.

Manufactured porous scaffolds can be then included in several types of macroscale bioreactors, like spinner flasks and rotating bioreactors, or in milliscale bioreactors, to reproduce interstitial perfusion. Spinner flasks and rotating wall vessels are two basic and inexpensive alternatives to static cultures and allow better nutrient transport and proliferation rate (Mccoy and O’brien, 2010; Yeatts and Fisher, 2011). They use convective flow to ensure the mixing of culture media around the 3D cellularized scaffolds. Spinner flasks are bioreactor systems made of a cylindrical container, with a stirring element at the bottom that ensures culture medium circulation and mixing (Figure 1A). These devices are suitable for mimicking native bone environment and study bone tissue formation and cellular function since they increase mass transport, shear stress, diffusion of nutrients. They also allow the removal of toxic products (Mccoy and O’brien, 2010; Yeatts and Fisher, 2011; Chen et al., 2013; Jin et al., 2014; Kedong et al., 2014; Song et al., 2016b; Zhang et al., 2017; Duan et al., 2018; Melke et al., 2018; He et al., 2019; Nadine et al., 2019; Tsai et al., 2019; Rubert et al., 2021; Van Beylen et al., 2021).

Rotating bioreactors offer major advantages over spinner flasks since they also control the supply of oxygen and exert low fluid shear stress and turbulence (Kimelman-Bleich et al., 2011; Weszl et al., 2012; Song et al., 2013; Shekaran et al., 2015; Song et al., 2016a; Demir et al., 2018; Westman et al., 2019; Daulbayev et al., 2020; Nokhbatolfoghahaei et al., 2020a; Nokhbatolfoghahaei et al., 2020b; Nokhbatolfoghahaei et al., 2020c). The most common rotating wall bioreactor is formed by two concentric cylinders: 1) the outer one is a culture chamber and accommodates the cellularized scaffold, submerged in culture medium, while 2) the inner cylinder is static and permits gas exchange (Figure 1B). However, in these rotation-based bioreactors, internal nutrient transport is limited to the outer compartment. This asymmetric spatial distribution of nutrients across the thickness of the scaffold may lead to the formation of a dense cell layer on the surface that, in turn, may cause an uneven distribution of fluid perfusion and shear stress and, ultimately, the formation of necrosis and impaired mineralization at the core of the construct (Mccoy and O’brien, 2010; Yeatts and Fisher, 2011). Active perfusion offered by perfusion bioreactors that directly pumps fluid through the cellularized structures overcomes these limitations.

Perfusion bioreactors can be considered as milli fluidics devices, with millimetric channel/culture chamber dimension, ranging from 1 to 10 mm, containing fluids volumes from 1 to 100 ml (Freed and Vunjak-Novakovic, 2002). These devices accurately mimic the effect of interstitial fluid flow into cellularized constructs by ensuring better environmental control, good mass transport, and ultimately, physical cellular stimulation (Mccoy and O’brien, 2010; Yeatts and Fisher, 2011). By comparing static and dynamic culture conditions, Tocchio et al. clearly demonstrated that three days-perfusion in a customized perfused bioreactor better avoided the formation of a necrotic core within a hydrogel-based porous scaffold, when compared to a cylindrical bulk agarose hydrogel placed in a static cell culture flask (Tocchio et al., 2015). Of course, here, it must be highlighted that the different porosity and composition of the scaffolds also play an important role on the formation of the necrotic core. These bioreactors foresee the use of pumps that perfuse the media through the scaffolds, either continuously or non-continuously, and that can be fully automatized. Several types of perfusion bioreactors have been tested so far, both commercial (i.e., U-Cup bioreactors, CELLEC Biotek AG108) and custom. These systems often have in common a basic functional module: a culture media reservoir, peristaltic pumps, a tubing circuit, and culture chambers (Figure 1C). In perfused bioreactors, fluid shear stress is finely tuned and applied directly by perfusing the cell-laden scaffold, or indirectly by applying external deformations, which cause perturbation of the media in the culture chamber (Mccoy and O’brien, 2010; Tseng et al., 2014; Wang et al., 2014). External deformation is obtained through cyclic compressive or tensile loading, torsion or ultrasound. In bone bioreactors, external deformation in the range of 1–30% has been obtained by applying compressive stress, and in the range 3–10% by applying tensile stress, both up to 21 days (Mauck et al., 2002; Kavlock and Goldstein, 2011; Liu et al., 2012; Petersen et al., 2012; Petri et al., 2012; Hao et al., 2013; Ramani‐Mohan et al., 2018). Torsion and ultrasound have been rarely used (Drapal et al., 2021). To date, however, a systematic investigation of different routes to apply shear stress on cells and scaffolds have not yet been carried out, which hinders a clear selection of the optimal route for different applications.

In conclusion, perfusion bioreactors have overcome the limits of previous macroscale systems, as they can reproduce bone microenvironment in a more accurate and controllable manner. As a demonstration, from 2010 to date, 81% of papers on bioreactors focuses on the effectiveness of perfused bioreactors in inducing bone cell differentiation and mineralization, while all the other strategies combined account for 9% (Web of science database). As a result, in the recent years, perfused bioreactors are replacing spinner flask and rotating vessel for 3D *in vitro* models of bone: after 2010, average 58 research papers/year with “Bone Perfus* Bioreactors” as key words vs seven paper/years with “Spinner Flask bone” and “Rotating vessel bone” as keywords (Web of science as reference database).

### 2.2 Microscale Models

**FIGURE 2:**
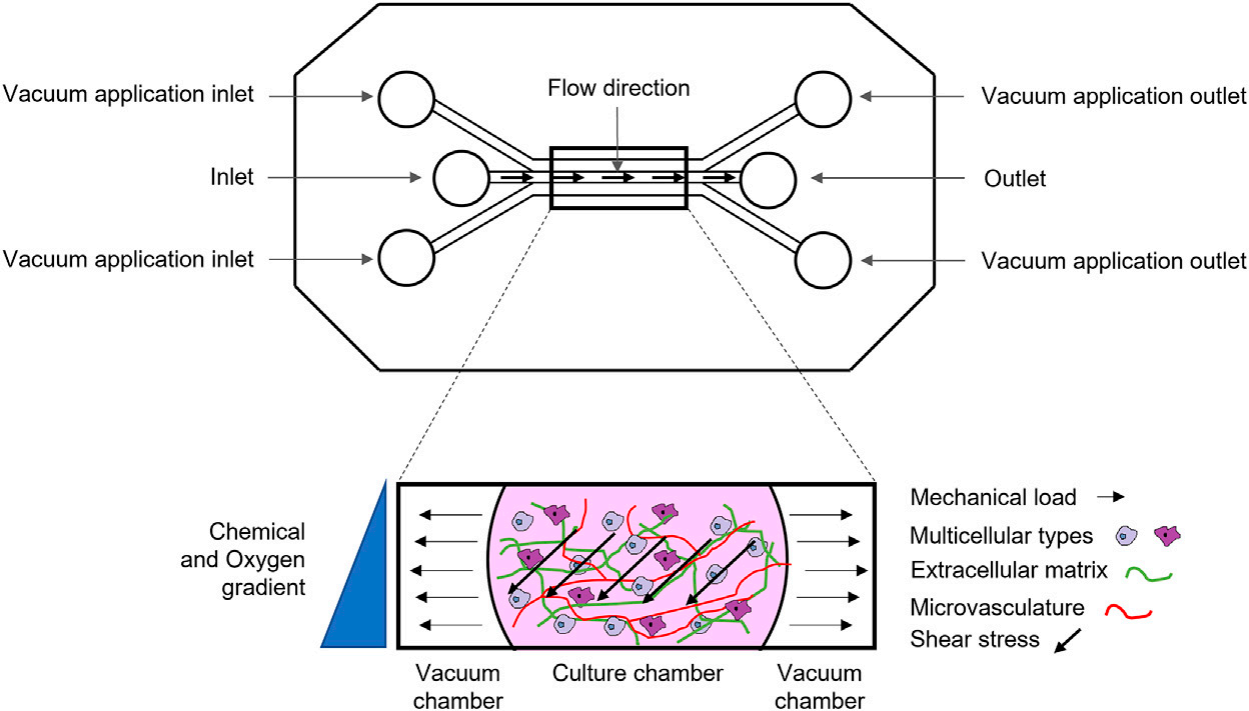
Schematic representation of a microfluidic device: micrometric channels exposed to multiple biochemical (e.g., chemical and oxygen gradient), biological (e.g., multicellular types, vasculature) and biophysical stimuli (e.g., shear stress, deformation).

Microfluidic systems, also called microfluidic bioreactors, are miniaturized bioreactor systems (Mestres et al., 2019)**,** with precise micrometric design to simulate nutrient delivery, paracrine communication, and specific crosstalk between multiple cell types, in either a 2D or a 3D microenvironment. Most importantly, microfluidic systems permit the application of mechanical stress at the microscale level, by tuning physiological flow and fluid shear stress (Figure 2) and more closely mimic the physiological stimuli that occur during cell-cell and cell-ECM interactions. Furthermore, microscale models do not lead to volumetric displacement of fluid that causes transient normal forces that may alter cell function, as it occur in macro and milliscale systems (Moraes et al., 2011). Finally, microfluidic systems allow for simultaneous refinement of biomechanical and biochemical properties to form a chemical gradient along with application of the physical stimulus (Mestres et al., 2019). Other key advantages of microfluidic devices are: 1) a significant reduction of the amount of reagents and cells to be used and 2) the possibility to quickly analyse large samples arrays, with high levels of precision and resolution and with live imaging (Sackmann et al., 2014). Here’s why, although the principle behind flow-induced shear stress is the same, microfluidic systems are more advantageous for mechanotransduction studies and analysis of complex phenomena, such as osteogenesis and angiogenesis, in real-time and at a high-resolution, than perfusion bioreactors. On the other hand, it should be underlined that these systems are still at their infancy in the bone research field and do not allow to reproduce the phenomena at the macro/milliscale. In particular, they fail to reproduce the complex 3D porous microarchitecture, composition and mechanical properties (e.g., Elastic modulus) of bone tissue (Rauh et al., 2011). By further analyzing the limitations of microfluidics, the standard materials used as ECM in these devices are thermoresponsive organic hydrogels (matrigel, collagen, fibrin) that can mimic or not the organic phase of bone and are easily injectable, but do not resemble bone inorganic composition and microarchitecture. Recent studies have paved the way to functionalize hydrogels with an inorganic phase (Liang et al., 2020), such as calcium phosphate (HA microbeads, HA nanoparticles, tricalcium phosphate TCP), borosilicate glass-ceramics, that, however, are still far from reproducing bone tissue characteristics (Sharma et al., 2021). Moreover, in micro-bioreactor, cell culture surface is very small (around 0.5–0.8 mm^2^) and can be seeded only with a few thousand cells. This feature can be considered as a pro and cons (Mattei et al., 2014)**.** On the one side, small volumes allow the insertion of patient-derived biopsies or cells inside the microfluidic chamber, which is crucial for the development of personalized therapeutic approaches (Arrigoni et al., 2017). On the other side, miniaturized organ-on-a-chip may be too simplistic in representing organ complexity (Mattei et al., 2014). In Table 1, we summarized pros and cons of perfusion device at the macro, milli and micro scale.

**TABLE 1:** Summary of pros and cons of perfusion devices at the macro, milli, and micro scale.

	*Pros*	*Cons*
***Macro scale***	Model bone porous microarchitecture Simulate mechanical properties of bone tissue Presence of convective flow to ensure the mixing of culture medium around the 3D cellularized scaffolds allowing for better nutrient/oxygen transport than static cultures	Limited medium transport inside the scaffold causing: • formation of a dense superficial cell layer on the surface • low supply of nutrients and oxygen to cells in the centre of the scaffolds • necrotic area at the core of the construct • uneven distribution of fluid shear stress, resulting in impaired mineralization in the inner part of the scaffolds Fail to model paracrine communication, cell-cell interaction, cell-ECM interaction at the microscale level	
***Milli scale***	Model bone porous microarchitecture Simulate mechanical properties of bone tissue Medium perfusion through the scaffold Accurately mimic the effect of interstitial fluid flow Wide range of mechanical loadings to be applied Possibility to model osteogenesis	Fail to model paracrine communication, cell-cell interaction, cell-ECM interaction at the microscale level No simultaneous application of biophysical and biochemical stimuli Fail to model complex biological phenomena that take place at the microscale, as angiogenesis and mechanotransduction Low resolution live imaging	
***Micro scale***	High resolution live imaging Reproducibility Accurately mimic the effect of interstitial fluid flow Simulate mechanical stress at microscale level Possibility to model paracrine communication, cell-cell interaction, cell-ECM interaction Possibility to combine multiple biochemical and biophysical stimuli to model complex biological phenomena (i.e., osteogenesis, mechanotransduction, angiogenesis) Reduction of the amount of reagents and cells Small number of cells allow insertion patient tissue biopsies, or cells isolated from biopsies, that are available in small quantities	Fail to mimic bone microarchitecture Fail to mimic mechanical properties of bone tissue Small range of materials available for in gel 3D cultures (i.e. injectable thermoresponsive hydrogels) Too simplistic in representing organ complexity due to small cell number	

## 3 Mimicking and Studying the Biological Features of Bone Microenvironment by Using Perfused Macro/Milli/Micro Bioreactors

Milli- and microscale perfusion systems enable *in vitro* recapitulation of bone microenvironment and complex mechanical, chemico-physical, and biological phenomena, such as the formation of oxygen and nutrients gradients, through physical barriers or through the formation of a vascularized network, osteogenic and angiogenic induction *via* mechano-stimulation, mechanotransduction, and the formation of an intercommunicating osteocyte 3D network. All micro environmental factors that are crucial for recapitulating bone biology and physiology in *in vitro* models and that we summarised in this chapter.

### 3.1 Oxygen Tension

*In vivo*, oxygen gradients occur naturally due to limitations in oxygen transport and metabolic consumption of oxygen by cells (Rexius-Hall et al., 2014) and different level of oxygen in the microenvironment may change cell behaviour and response. Furthermore, oxygen levels are specific to different cell types or components of bone (Hirao et al., 2007; Volkmer et al., 2008; Volkmer et al., 2010; Volkmer et al., 2012; Inagaki et al., 2017; Lee et al., 2017; Stegen et al., 2018; Urdeitx et al., 2020). For instance, in bone tissue, different compartments are characterized by different levels of oxygen tension (pO_2_), which is high in the periosteum, low in cortical bone, and even lower in bone marrow, despite its very high vascular density (Spencer et al., 2014).

**FIGURE 3:**
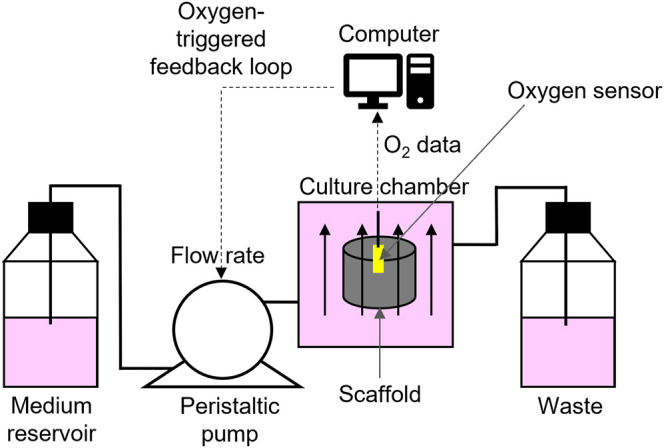
Schematic view of the oxygen-triggered feedback mechanism: a computer controlled peristaltic pump drives fresh medium from the reservoir through the perfusion bioreactor into the waste reservoir. The oxygen sensor (yellow) constantly senses the oxygen concentration in the centre of the scaffold and sends data to the computer, which controls the pump speed.

*In vitro*, perfusion ensures the transport of oxygen across the cellularized construct, both at the milli- and microscale. However, obtaining a fine control of the oxygen level in *in vitro* models is not trivial. In milliscale systems, optical oxygen micro sensors can be inserted in the cell-laden scaffold to send pO_2_ data, through an oxygen-triggered feedback mechanism. Data are collected by a computer that controls a syringe pump that, according to the type of input received by the computer, adjusts the perfusion rate by activating or stopping the fluid flow (Volkmer et al., 2008; Volkmer et al., 2010; Volkmer et al., 2012). Therefore, these systems are suitable for setting a predefined pO_2_ in the culture chamber, but are unsuitable to create oxygen gradients (Figure 3). As for microfluidic systems, different devices have been developed to study the effect of O_2_ tension on cellular behaviour, including the response to drug treatments. They can be engineered to allow a tight control of cell exposure to given oxygen levels, either one single oxygen level or multi-condition oxygen levels. This is obtained by the use of off-chip computer-controlled gas mixers, flow of oxygen scavenging chemicals, or on-chip gas mixer layouts (Rexius-Hall et al., 2014). Therefore, microfluidics appear more promising to achieve controlled oxygen gradients than the milliscale devices. These technologies are now well established and prospectively will be more extensively applied for tissue modelling. To date, however, very few papers are available.

### 3.2 Vascularization

For applications of bone tissue constructs in tissue engineering, it is already clear that the lack of vascularization may result in functional and physical failure upon implantation due to cell necrosis (Bandaru et al., 2020; Harvestine et al., 2020; Hann et al., 2021). Bone vascularization is critical to ensure bone cell survival since it allows the perfusion and delivery of fundamental cell nutrients and the removal of waste products of cell metabolism and this is the reason why inclusion of vascularized structures also in perfused bioreactors is a very active field of investigation.

#### 3.2.1 Reproducing and Studying Vascularization in Macro and Milliscale Bioreactors

Recently, perfused bioreactors are often designed to include a vessel compartment, seeded with endothelial cells (e.g., human umbilical vein endothelial cell, hUVEC), and aimed at recreating those microenvironmental conditions that can promote both angiogenesis and osteogenesis (e.g., biomimetic matrix, perfusion, mechanical cues, biological cues) (Bandaru et al., 2020; Harvestine et al., 2020; Hann et al., 2021). As an example, Sung Yun Hann et al. obtained a perfusable vessel with an inner diameter of 800 µm by using an FDM-printed polyvinyl alcohol (PVA) sacrificial template within a stereolithography-printed biomimetic bone tissue construct (Hann et al., 2021). The large vessel channel was then seeded with endothelial cells and perfused with cell culture media, by means of a digital peristaltic pump at a flow rate similar to those of *in vivo* vascularized bone microenvironment (5 ml/min).

Beside 3D printing, 3D bioprinting is another emerging technology to obtain vascular networks and allows a better control over both cell distribution and scaffold size, shape, and architecture. By this technique, it is possible to deposit material and cells at one same time (Jia et al., 2016; Datta et al., 2017). However, this approach is still at its early stages in the orthopaedic field and far from its clinical use, since several parameters need to be carefully adjusted. Among these, cell density, resolution of perfusable channels, and bioink composition should be optimized to obtain angiogenic sprouting and neovascularization and their tuning is a very challenging task (Richards et al., 2017; Vidal et al., 2020; Hann et al., 2021). Regarding the bioink composition, gelatin methacrylate (GelMa) is one of the most widely used natural-based hydrogels as it improves cell adhesion and growth of the vascular network (Yue et al., 2015; Gao et al., 2019) and resembles the composition of the organic part of bone made of collagen. To further increase biomimicry, GelMa can be functionalized with ceramic fillers, such as hydroxyapatite nanoparticles, to simulate the inorganic component of bone tissue, modulate the surface roughness of the scaffold, increase the surface area for endothelial cell attachment, and finally, enhance mechanical competence of the scaffold (Hann et al., 2021). To this aims, along with the most widely used HA, several other bioactive and non-bioactive ceramics have been proposed, including biphasic calcium phosphate (BCP), TCP, and bioactive glass (Hann et al., 2021). Regarding the cell histotype to be used to obtained vascularised constructs through bioprinting, many scientific works have emphasized the importance of cells of the mesenchymal lineage, both in stabilizing newly formed capillaries, like MSCs differentiated into pericytes and fibroblasts, and in inducing a proangiogenic stimulus, especially under dynamic culture conditions. As an example, several authors demonstrated that the co-implantation of HUVEC and MSCs facilitates the formation of long-lasting functional vasculature, and stabilizes the capillary networks, thanks to the secretion of VEGF, platelet derived growth factor AA (PDGF-AA), platelet derived growth factor BB (PDGF-BB), and TGF-β (Koike et al., 2004; Au et al., 2008; Stratman and Davis, 2012; Wanjare et al., 2013). For this reason, both human MSCs and endothelial cells have been often included in the development of bioprinted bioreactors that mimic vascularized bone (Oskowitz et al., 2011; Rasmussen et al., 2011; Barclay et al., 2012; Edwards et al., 2014; Temple et al., 2014; Wang et al., 2014; Kehl et al., 2019; Nasser et al., 2019; Bandaru et al., 2020; Chiesa et al., 2020; Harvestine et al., 2020; Hann et al., 2021; Winkler et al., 2021). However, the role of mechanical stimulation and dynamic culture conditions in modulating MSCs pro-angiogenic activity is still relatively unexplored (Bandaru et al., 2020; Harvestine et al., 2020), with a few exceptions, like data presented by Praveen Bandaru et al. showing that the application of mechanical strains by cyclic compression on matrices-embedded MSCs induces the secretion of VEGF and increases hUVEC tubulogenesis (Bandaru et al., 2020).

Although biofabrication and additive manufacturing approaches are very promising, it is worth remembering that these techniques fail to fully replicate the complex hierarchical 3D microarchitecture of the vascular network since it is hard to reproduce the finer vascular structures (at the micrometre scale), like capillaries and venules. An intriguing solution to this technical limitation has been proposed by few authors who fabricated two parallel endothelialized millimetric fluidic channels, with a fibrin–endothelial-cell mixture, through a technique based on sacrificial template materials (Ozbolat, 2015; Arrigoni et al., 2017). The developed device was treated with pro-angiogenic stimuli to promote neogenesis of adjacent capillaries from endothelial cells layered on the millimetric vessel. We believe that the combination of different additive manufacturing techniques (such as 3D printing and electrospinning) will permit to solve this need. Indeed, the combination of 3D printing and electrospinning is increasingly used (Lee et al., 2009; Chang et al., 2017; Giannitelli et al., 2018). This approach is opening new perspectives in the development of 3D microfluidic models, because it allows to: 1) mimic the composition and fibrous morphology of the ECM, 2) tune the morphological characteristics of the chambers or and/or 3) regulate the flux in the channels (Giannitelli et al., 2018). Applications have been proposed in several fields, including the study of human bone marrow-derived MSCs under different perfusion conditions and surface characteristics (Lee et al., 2009). It is therefore very likely that it will also be used in the near future for the study of bone tissue and microenvironment.

#### 3.2.2 Reproducing and Studying Vascularization in Microscale Bioreactors

Microfluidic devices are very amenable support to assess the angiogenic potential and its coupling to osteogenesis in a bone-like microenvironment. Three are three basic factors that should be considered for their development to study angiogenesis in bone: 1) the choice of biomaterials, 2) the type of stimuli to be included and 3) the device’s design. As biomaterials, the inclusion of HA within the hydrogel (i.e., fibrin or collagen) injected in the channel can greatly improve the device performance. Indeed, HA-rich environment is stiffer than the pure hydrogel and it positively influences vessel lumen formation, in terms of sprout length speed, number of sprouts and lumen diameter (Jusoh et al., 2015). Furthermore, the presence of specific molecules, such as growth factors, may also influence blood vessel formation. The addition of lung fibroblasts to the system, for example, enriches the hydrogel with fibroblast-derived chemotactic and pro-angiogenic factors, such as VEGFs and ECM proteins that are critical in inducing hUVEC morphogenesis. Beside ECM composition and biochemical stimuli, mechanical stimulation also regulates the osteo-angio crosstalk in the context of angiogenesis. Liu et al. demonstrated that a conditioned media of osteoblast cultures, exposed to shear stress, enhances endothelial cell proliferation and migration (Liu et al., 2016). The most comprehensive example of a study on the use of a microfluidic system mimicking bone and including angiogenesis is the one proposed by Sano E. et al., who combined angiogenesis and anastomosis (Sano et al., 2018). The bone microenvironment was recapitulated by culturing multicellular spheroids (osteo-differentiated MSCs, endothelial cells and fibroblasts) embedded into a hydrogel (fibrin/collagen) within the central channel of a microfluidic device, subjected to fluid flow. Side channels were then seeded with endothelial cells to form tubular structures, so that angiogenic sprouts from the cell spheroids and microchannels were anastomosed to form a 3D vascular network. This study clearly demonstrated that it is also possible to develop models with a very high degree of complexity and that can be used, for the future, as a prototype for fully customized patient-specific models.

On the whole, both at the milli and microscale, the reproduction of bone microenvironment, and more specifically, the coexistence of endothelial cells and cells of the mesenchymal lineage (i.e., MSC), mechanical cues (i.e., shear stress) and stiff osteomimetic matrix (i.e., HA-enriched hydrogel), can be modulated and sued advantageously to obtain *in vitro* vascularised bone models.

### 3.3 Osteogenic Cells

Direct axial loading on bone and cartilage *in vivo* results in compressive forces that, in turn, generate pressure gradients and interstitial fluid flow. It has been already well established that osteoblasts and MSCs directly respond to shear stress by increasing the expression of the early and late osteoblastic markers and calcium deposition. The use of perfused bioreactors is extremely suitable to study these types of mechanic-biological stimulations. The most commonly applied mechanical stimuli to obtain shear stress *in vitro* in perfused bone models is compression; also tension, torsion and ultrasound are widely investigated although with aims other than studying osteogenic differentiation. In this chapter, we will discuss on how milli and microbioreactors have been used to study the effect of fluid perfusion on osteogenic differentiation.

#### 3.1.1 Studying Osteogenesis in Macroscale and Milliscale Bioreactors

Interstitial fluid flow modulates osteogenesis and can be recapitulated *in vitro* at the milliscale level through the generation of fluid shear stress or through the application of mechanical strains (Alfieri et al., 2019; Ramani-Mohan et al., 2018), by using both commercially available and custom-made perfusion bioreactors (Tseng et al., 2014; Kleinhans et al., 2015; Kanda et al., 2016; Beşkardeş et al., 2018; Burgio et al., 2018; Ramani-Mohan et al., 2018; De Luca et al., 2020). U-Cup bioreactors are the most widely used among those offered by the market, as they effectively perfuse media into the scaffold through a pump system. The core of the system is the perfusion cartridge which houses the scaffold that is sealed to avoid that the medium fluxes around its surface, so that the media is perfused directly through the scaffold pores (Yeatts and Fisher, 2011). Custom-made perfusion bioreactors offer the important advantage to allow computational simulation of the flow during the device’s design phase, prior to fabrication. As elegantly shown by Kleinhans et al. (2015), Ramani-Mohan et al. (2018) and Schmid et al. (2018), the device customization makes it possible to predict the fluid distribution and fluid shear stress distribution across porous structures and define the device geometry that shall be optimal for nutrients, metabolites and oxygen diffusion, according to the specific aim. In customised devices, shear stress can be applied directly or indirectly, as previously discussed in chapter Section 2.1. In particular, external deformations to indirectly induce shear stress can be obtained by external actuators that are integrated within the bioreactor system (Matziolis et al., 2011; Petersen et al., 2012; Ramani-Mohan et al., 2018). As an example, Ramani-Mohan R. et al. developed a strain-responsive construct including immortalized MSCs in a porous PLLA-co-PCL scaffold, subjected to controlled mechanical culture conditions (Ramani-Mohan et al., 2018). Briefly, they coupled a perfusion bioreactor (connected to an external peristaltic pump) to a linear motion device, thus obtaining both media perfusion (1.6 ml/min) and uniaxial compression cycles (1–2% deformation at 1–2 Hz), respectively. The combination of perfusion-induced fluid shear stress (1.73 × 10^–4^ Pa) and compression of the scaffold induced a calcification activity, as assessed by alizarin red staining, analysis of mRNA expression, analysis of regeneration and bone remodelling-related osteogenic genes (SPARCON, Secreted Phosphoprotein 1 (SPP1), Collagen Type I Alpha 1 Chain (Col1A1), RUNX2, ALPL, BMP-2). Both perfusion-induced fluid shear stress and compression of the scaffold had an impact on the expression of osteogenic markers and on calcification. The authors also developed a computational model to estimate the profile of the perfusion flow that modelled the dynamic fluid shear stress exerted on the cyclically loaded scaffolds and confirmed that deformation strain was the predominant stimulus toward the osteogenic lineage. In addition to the work of Ramani-Mohan et al., many other studies have been published on the effect of dynamic loading in increasing extracellular matrix mineralization and deposition, or on the upregulation of the expression of osteogenic markers, including collagen I, bone morphogenic protein-2 (BMP-2), osteonectin (ON), osteocalcin, osteopontin, Runt-related transcription factor 2 (RUNX2), or ALP (Ramani-Mohan et al., 2018; Hoffmann et al., 2005; Tsai et al., 2014; Petersen et al., 2012).

Finally, it is worthwhile to mention that the addition of osteomimetic properties to the scaffolds included in bioreactors may further increase the impact of perfusion and mechanical cues on bone osteogenesis. Previous studies showed that the addition of HA to the scaffold is a valuable strategy to reproduce bone ECM as it is very similar to the major inorganic component of natural bone. HA can be used alone (Matziolis et al., 2011; Tseng et al., 2014; Burgio et al., 2018; De Luca et al., 2020) or as a filler in composite materials (Volkmer et al., 2008; Volkmer et al., 2012; Beşkardeş et al., 2018; De Luca et al., 2020). As an example, Burgio et al. produced porous discs (10 mm diameter, 4 mm thick) from HA powder with a 3D-printing system, with an internal porosity of 61%, and an internal pore dimension ranging from 300 to 600 μm (macropores), and from 10 to 15 µm (micropores) (Burgio et al., 2018). Besides, Elias Volkmer et al. produced a composite scaffold of nHA dispersed in a biocompatible polyurethane-based polymer (Volkmer et al., 2012), by using dispense-plotting. This rapid prototyping technique allows the extrusion of ceramic paste through a nozzle by pressurized air to obtain 3D interconnected structures with controlled porosity. The HA-enriched porous scaffolds, seeded with MSCs and subjected to dynamic perfusion, showed excellent homogeneity of cells distribution and high expression of key factors of osteogenic differentiation (i.e., RUNX2, ALP, collagen I and osteocalcin) (Volkmer et al., 2012; Burgio et al., 2018). An another example is offered by the study of De Luca at al. showing the effects of ceramic component in the bone-like scaffold, made of poly-l-lactic-acid (PLLA)/nano-hydroxyapatite (nHA) composite, and of the combination of a biomimetic scaffold and active perfusion on the osteogenic differentiation of MSCs (De Luca et al., 2020). In particular, the authors observed that the PLLA/nHA composite scaffold induced the upregulations of osteogenic markers in MSCs, that, however, was further enhanced, like RUNX2, ALP, SPP1 and SRY-Box Transcription Factor 9 (SOX9), and coupled to calcium nodule formation, when physical stimulation was also applied (De Luca et al., 2020).

In conclusion, perfusion bioreactors, both commercial and custom, are particularly suitable to model bone and for studying the induction of osteogenesis. Perfusion enhances the expression of specific osteogenic markers through the induction of physiologically relevant shear stress. The expression of these markers is further increased when external mechanical loading is applied (e.g., cyclic compression) and when HA particles are added to the scaffold to mimic the bone inorganic component.

#### 3.1.2 Studying Osteogenesis in Microscale Bioreactors

To date, several microfluidic devices have been used to investigate on the osteogenic potential of MSCs when custom-made miniaturized geometry and fluid shear stress are combined. Results obtained by these studies mostly confirmed the findings of macro and milliscale experiments. In microfluidic setups, mechanical cues are applied through perfusion or by external stimuli (Mccoy and O’brien, 2010; Tang et al., 2010; Yourek et al., 2010; Kim and Ma, 2012; Stavenschi et al., 2017). An example of perfusion-induced shear stress in a microfluidic bioreactors is proposed by Junho K. and Ma T, who investigated MSCs properties, including the expression of osteogenic markers, under two perfusion flow conditions, around and through the construct (Kim and Ma, 2012). The device was formed by four chambers, two of which operated under the parallel flow and two under transverse flow. Shear stress induced by the parallel flow was more effective than shear stress induced by transverse flow in enhancing ALP expression and mineralization. In fact, even though shear stress, derived from transverse flow, stimulated cell proliferation, through the convective flow also removed ECM proteins and secreted growth factors (e.g., fibroblast growth factor), ultimately affecting osteogenic differentiation.

As another example with a higher degree of complexity, Gao X. et al. designed a membrane-based microfluidic chip to study the effect of shear stress on proliferation and differentiation of MSCs, when it is induced by cyclic tensile stress on a cell membrane (Gao et al., 2011). The chip was composed by three polydimethylsiloxane (PDMS) layers: a top layer containing cell culture channels, a bottom layer containing gas control channels, and a middle elastic membrane, sandwiched between the two layers, irreversibly sealed by oxygen plasma. The cyclic tensile stress was generated by the PDMS membrane deformation which, in turn, was induced by pulsed negative pressure applied to the gas control channel. The degree of tensile stress was directly correlated to the degree of membrane deformation. Three classes of membrane deformation was studied, high (>3.5%), moderate (2.8–3.2%) and low (≈2.2%), but only high deformation was effective in significantly increasing ALP expression. However, also moderate deformation induced MSCs osteogenic differentiation to an extent similar to those obtained with differentiating medium. Furthermore, the authors explored whether the tensile stress could affect adipogenic differentiation, that showed an opposite trend in respect to osteogenic differentiation under higher stress.

The potential of dynamic hydraulic compression to induce fluid shear stress was investigated by Sang-Hyug Park et al. that, by using a microscale fluidic device, applied a dynamic hydraulic compression simultaneously on two different cell types, human bone marrow- and adipose-derived MSCs (Park et al., 2012). Briefly, to generate hydraulic compressive force, the microfluidic device was connected to a pneumatic control setup, whereas pressure was controlled with a fast-switching solenoid valve driven by electric circuit with pulsatile signal. Pulsatile pressure was applied into the air chamber inside the microfluidic device, causing the deformation of a PDMS membrane placed on top of the culture chamber. The membrane, in turn, transmitted the stimuli to the osteogenic media and to the cells. Cells were cultured on the bottom of the cell culture chamber and periodically exposed to cyclical loading (10 min every 12 h for 7 days). This study demonstrated that dynamic hydraulic compression (1 Hz, 1 psi) increases the production of osteogenic matrix components (i.e., bone sialoprotein, osteopontin, collagen type I) and boosts integrin expression. Bone marrow-derived MSCs were more sensitive to mechanical stimulation and more prone towards osteogenic differentiation than adipose-derived MSCs. Finally, an example of a very innovative approach was proposed by Lembong J. et al. who obtained a spatially patterned proliferation and differentiation of MSCs by combining perfusion to substrate micropatterning (Lembong et al., 2018). Briefly, they developed a 3D-printed fluidic chamber for the dynamic culturing of MSCs that were seeded on an array of cylindrical pillars. Under these conditions, the authors obtained a higher osteogenesis differentiation in the region near the pillars.

In conclusion, microfluidic devices are advanced tools that exploit custom geometry, micro-sized channels that can also be patterned, and deformable/non deformable structures, to finely tune perfusion (different flow conditions) or mechanical stimuli (e.g., shear stress, tensile stress, dynamic hydraulic compression) to modulate and study osteogenic differentiation.

### 3.4 The Osteocytes Network

Osteocytes are the most differentiated form of osteoblasts and the most common type (90–95%) of bone cells with a fundamental role in the regulation of bone and mineral homeostasis. *In vivo,* osteocytes reside in lacunae, which are interconnected microscale spaces 20–30 µm apart from each other (mouse bone). The intercellular dimension is important in cell–cell signalling for osteocyte process growth and mechanotransduction sensitivity (Gu et al., 2015). Nonetheless, osteocytes are quite rarely included in preclinical models, and even less in perfusion devices (average of three published papers per year since 2015, Scopus Database) (Mullen et al., 2013; Bellido, 2014; Choudhary et al., 2018; Sun et al., 2018). The main reason is possibly the difficulty of isolating osteocytes from the mineralized bone and recreating a 3D cell microenvironment that can mimic the lacunar-canalicular structure of bone tissue and the interstitial fluid flow (Webster et al., 2013; Sun et al., 2018), and the formation of an osteocytic network, all crucial for the osteocyte physiology. Thus, the use of 3D perfused devices may be particularly advantageous for the study of this specific bone cell. Form one side, 3D microfluidic device may allow the use of multicellular models, like osteocytes cultured with other cells of the bone microenvironment (e.g., osteoblasts and/or osteoclasts), to study the role of the osteocytic network on bone homeostasis and analyse its ability to induce osteoblastogenesis and osteoclastogenesis (Middleton et al., 2017; George et al., 2018). On the other side, osteocytes are mechanosensors, sensing to different kinds and extents of mechanical load, and reacting through the regulation of bone homeostasis (Brown et al., 2013; Bellido, 2014; Florencio-Silva et al., 2015; Terpos, 2015; Bellido et al., 2019), and thus, microscale bioreactors are very useful for study osteocyte-mediated mechanotransduction.

As an example, by the use of milli scaled devices, co-culture experiments showed that fluid shear stress induces the release of factors by osteocytes that affect osteoblasts and osteoclasts activity and modulate osteoblasts proliferation and differentiation, also through the release of nitric oxide (Vezeridis et al., 2006; Hoey et al., 2011; Wittkowske et al., 2016). Similarly, the application of compressive stress (5 min, 10 Hz, 2.5 N) on a 3D co-culture model, including osteocytes that were embedded in a Type I collagen gel, and osteoblasts that were cultured on top of the hydrogel, induced the formation of an osteocytic network within the collagen and increased collagen production by osteoblasts (Vazquez et al., 2014).

At the microscale, a larger number of studies has been published. Microfluidic devices incorporating osteocytes are usually focused onto three main aspects: 3D cell distribution, biomaterials, and mechanical stimuli (LA and Alam; Li et al., 2008; Gu et al., 2015; Wei et al., 2015; Xu et al., 2016; Middleton et al., 2017; George et al., 2018; Sun et al., 2018). To recapitulate this crucial structure *in vitro*, Gu et al. developed a cell construct in which osteocyte cell bodies were located into the interstitial spaces between BCP microbeads (ø 20–30 µm) (Gu et al., 2015). To recreate a bone-like microenvironment, other authors used collagen-based hydrogels and ceramics fillers that favoured the upregulation of osteocyte specific genes, such as Sost gene, a key osteocyte-specific marker for mechanotransduction (Wei et al., 2015; Middleton et al., 2017; Sun et al., 2018), or collagen-coating chamber that was effective in the maintenance of the osteocyte phenotype (Wei et al., 2015). Besides the composition of the ECM, the key feature that makes the model particularly suitable for reproducing the *in vivo* counterpart is the mechanical-induced shear stress that, as explained above, is mandatory for the characterization of osteocytes behaviour and features in bone homeostasis and pathology (LA and Alam; Li et al., 2008; Middleton et al., 2017; George et al., 2018). The most representative example is the device developed by Qiaoling Sun et al. who assembled an osteo-like structure mixing collagen-coated biphasic calcium phosphate microbeads (68% of HA and 32% of β-TCP) with MLO-A5 cells. Furthermore, cells were exposed to a cyclic compression-induced shear stress, obtained by cell chamber pressurization (Sun et al., 2018).

In summary, in fluidic bioreactors, osteocytes can form a 3D network to communicate with the other bone cells, and are extremely sensitive to mechanical loading, and respond to such external stimuli by releasing soluble factors which, in turn, control bone homeostasis. As a result, contrary to static models, microfluidics have the potential to mimic the dynamic osteocyte microenvironment (i.e., shear stress, ECM), paving the way to the development of *in vitro* reliable bone model, to fully understand the function of osteocytes in physiological and altered states.

## 4 Perfused Bioreactors for Drug Screening of Anabolic and Anti-catabolic Drugs

The potential to develop *in vitro* complex bone models makes perfusable milli- and microscale bioreactors promising tools to create reliable drug screening platforms for bone-related diseases. Nowadays, these platforms are extensively studied for the screening of anti-cancer treatments, which are by far the most widely explored in bioreactors. However, also anabolic and anti-catabolic drugs could be considered for a pre-screening of treatments for bone metabolic disorders (e.g., osteoporosis) and are now under investigation. Anabolic and anti-catabolic drugs increase bone strength and reduce fractures by favouring the synthesis of bone or by slowing bone resorption, respectively (Riggs and Parfitt, 2005; Lyritis et al., 2010; Ripamonti, 2017).

A few recent research works based on milliscale bioreactors have demonstrated that the combined effect of perfusion and anabolic drugs (e.g., 2-chloro-5-nitrobenzanilide, a PPARγ inhibitor (GW9662), hydrogen sulphide, parathyroid hormone) can enhance collagen deposition and bone mineralization (Grant et al., 2016; Liu et al., 2018; Gambari et al., 2019; Mondragon et al., 2020; Liu et al., 2021). Mondragon et al. showed that perfused cultures of MSCs on lyophilized bovine collagen type I scaffolds upon which Mg-doped HA nanocrystals nucleated during collagen fibrils self-assembly and treated with GW9662, led to an increased scaffold mineral density and compressive modulus (Mondragon et al., 2020). Contrary to milliscale, at the microscale this topic is completely unexplored. Also, the use of anti-catabolic drugs in perfused bone models is at its early stages, with only two papers published, for milli and microscale models, respectively (Xu et al., 2016; Naqvi et al., 2020). Both papers focus on the effect of drug treatments on the mechanoresponsiveness of mechanically stimulated osteocytes and osteoblasts (e.g., fluid shear stress, hydrostatic pressure). More in details, at the milliscale, Naqvi S. M. et al. investigated whether oestrogen deficiency affects the differentiation of mechanically stimulated osteoblasts towards an osteocytic lineage, and studied the osteoblast mineralization activity, and the physiological paracrine signalling between osteoblasts and osteoclasts during bone resorption (Naqvi et al., 2020). At the microscale, to test the effect of zoledronic acid on osteolysis, Liangcheng Xu mimicked a more complex and complete bone microenvironment during physiological bone resorption by recreating the interaction between osteocytes and osteoclasts, cultured under physiologically interstitial fluid shear stress. As a result, they demonstrated that the addition of the anti-osteolytic drug caused a significant decrease in osteoclast differentiation in the system (Xu et al., 2016).

## 5 Conclusion

The ever-increasing life expectancy has led to an augment of the portion of the elderly population, more frequently subjected to musculoskeletal-related morbidities. Therefore, the study of musculoskeletal disorders and the development of treatments is of paramount importance in clinical research. However, reproducing bone *in vitro* is a challenging task due to its complex composition, 3D structure and function. Perfused models, both at the micro, milli and macro-scale, represent an innovative field of research as they bear crucial features that can overcome the limitations of traditional cell culture methods, such as over-simplification of the bone microenvironment, as they allow to simulate interstitial fluid flow and recapitulate flow-dependent biological processes. Fluid flow can be reproduced by different systems, with different degrees of complexity. To this regard, milliscale bioreactors take advantage of perfusion systems to model bone tissue at relevant physiological size. Bioreactors can be distinguished in two categories: macroscale traditional bioreactors, as spinner flask and rotating wall bioreactor, and milliscale bioreactors. The latter allows a controlled perfusion through the cell construct, thus better mimicking physiological interstitial fluid flow. These systems are particularly suitable to model the differentiation process of the osteogenic lineage, since dynamic perfusion enhances the expression of osteogenic markers, through the induction of physiologically relevant shear stress. Another and quite recent and innovative way to develop bioreactors at the milliscale is offered by biofabrication techniques that are particularly promising for the study of vasculogenesis. However, yet, complex hierarchical 3D microarchitecture of vascular network cannot be recapitulated. Besides, macro/milli size are not properly adequate to study fine cell-cell communication. On the opposite, microbioreactors based on microfluidic techniques allow the control and combination of multiple biochemical and biophysical stimuli, and the observation of biological phenomena in real-time and at a high-resolution, including intercellular paracrine communication, direct cell-cell interaction, and specific crosstalk between multiple cell types. In these devices, cells can be cultured in a finely controlled 3D microenvironment and under physiological flow and fluid shear stress, and biological activities at the microscale, such as osteogenesis, angiogenesis and mechanotransduction, can be more easily analysed. Furthermore, the micro-size is an advantage in terms of cell number and reagent volume. However, like for milliscale bioreactors, microfluidic devices present some limitations since they cannot reproduce the complex microarchitecture and mechanical properties of bone tissue.

In conclusion, perfused models are promising tools to investigate complex 3D tissues and their microenvironment and are likely the key to build more realistic *in vitro* models of bone for studying and understanding bone pathophysiology, and for the identification of novel anabolic or anti-catabolic drugs. However, a thorough survey of literature revealed that one has to still wait for a coordinated combined system that can adequately model bone biology and physiology as each of these perfused devices recreates a single feature and none of them can be considered as “bone-like” complete model.

## Footnotes

1 WHO2018, https://www.who.int/news-room/fact-sheets/detail/ageing-and-health.

## Abbreviations

2D, two dimensional; 3D, three dimensional; ALP, alkaline phosphatase; ASCs adipose tissue-derived stem cells; BCP, biphasic calcium phosphate; BMP-2, bone morphogenic protein-2; CollA1, Collagen Type I Alpha 1 Chain; ECM, extracellular matrix; FDM, Fused deposition modelling; GelMa, Gelatin methacrylate; GW9662, 2-chloro-5-nitrobenzanilide, a PPARγ inhibitor; HA, hydroxyapatite; hUVEC, human umbilical vein endothelial cell; MSCs, mesenchymal stromal cells; ON, osteonectin; PDGF-AA, platelet derived growth factor AA; PDGF-BB, platelet derived growth factor BB; PDMS, polydimethylsiloxane; PLLA, poly (l-lactic acid); PLLA-co-PCL, poly (l-lactide)-co-poly (ε-caprolactone); pO2, Oxygen tension; PVA, polyvinyl alcohol; RUNX2, Runt-related transcription factor 2; SOX9, SRY-Box Transcription Factor 9; SPP1, Secreted Phosphoprotein 1; TCP, tricalcium phosphate; TGFβ, transforming growth factor beta; VEGF, Vascular Endothelial Growth Factor.
